# Aldehyde Dehydrogenase 2 Protects Against Lipopolysaccharide-Induced Myocardial Injury by Suppressing Mitophagy

**DOI:** 10.3389/fphar.2021.641058

**Published:** 2021-05-07

**Authors:** Wenqing Ji, Tiantian Wan, Fang Zhang, Xiaomei Zhu, Shubin Guo, Xue Mei

**Affiliations:** ^1^ Emergency Medicine Clinical Research Center, Beijing Chao-Yang Hospital, Capital Medical University, Beijing, China; ^2^ Beijing Key Laboratory of Cardiopulmonary Cerebral Resuscitation, Beijing, China

**Keywords:** ALDH2, lipopolysaccharide, myocardium, mitophagy, PINK1/parkin, oxidative stress

## Abstract

Sepsis is defined as life-threatening organ dysfunction caused by a dysregulated host response to infection. Sepsis-induced circulatory and cardiac dysfunction is associated with high mortality rates. Mitophagy, a specific form of autophagy, is excessively activated in lipopolysaccharide-induced myocardial injury. The present study investigated whether aldehyde dehydrogenase 2 (ALDH2) regulates mitophagy in sepsis-induced myocardial dysfunction. After lipopolysaccharide administration, cardiac dysfunction, inflammatory cell infiltration, biochemical indicators of myocardial cell injury, and cardiomyocyte apoptosis were ameliorated in mice by ALDH2 activation or overexpression. In contrast, cardiac dysfunction and cardiomyocyte apoptosis were exacerbated in mice followed ALDH2 inhibition. Moreover, ALDH2 activation or overexpression regulated mitophagy by suppressing the expression of phosphatase and tensin homolog-induced putative kinase 1 (PINK1)/Parkin, by preventing the accumulation of 4-hydroxy-trans-nonenal. Conversely, ALDH2 inhibition promoted the expression of LC3B by increasing 4-hydroxy-trans-2-nonenal accumulation. Consequently, ALDH2 may protect the heart from lipopolysaccharide-induced injury by suppressing PINK1/Parkin-dependent mitophagy.

## Introduction

The cardiovascular system is a major target of sepsis, which is a systemic inflammatory response that can induce organ dysfunction (Annane et al., 2005) and is the leading cause of in-hospital mortality (Liu et al., 2014). Septic shock (Kumar et al., 2000; Court et al., 2002; Levy and Deutschman, 2004; Rabuel and Mebazaa, 2006; Zanotti-Cavazzoni and Hollenberg, 2009) and myocardial dysfunction are closely related to the increased mortality of sepsis patients (Landesberg et al., 2012). To date, there is no effective treatment for sepsis-related cardiac dysfunction. Therefore, an effective method is urgently needed to treat this disease.

The mechanisms underlying sepsis-induced myocardial damage are related to a number of different processes, including mitochondrial dysfunction, oxidative stress, cardiomyocyte apoptosis, direct effects of bacterial toxins, calcium dyshomeostasis, and impaired *β*-adrenaline signaling (Suffredini et al., 1989; Chen et al., 2003; Drosatos et al., 2015; Hobai et al., 2015; Zhao et al., 2016). Among them, mitochondrial dysfunction plays a dominant role in the development and progression of cardiac dysfunction in sepsis (Chen et al., 2003).

Autophagy, which functions in physiological and pathological conditions, has prominent roles in cellular degradation through the lysosomal degradation pathway (Cuervo, 2004). Autophagy is a double-edged sword that can inhibit but also promote cell apoptosis. Autophagy can remove cytosolic proteins and organelles, such as mitochondria, and ultimately inhibit apoptosis after lipopolysaccharide (LPS) administration. However, excessive autophagy can also lead to cell death by promoting apoptosis (Scott et al., 2007; Hsieh et al., 2009). Similarly, autophagy can exert diverse effects during myocardial ischemia and reperfusion; autophagy can protect the myocardium during ischemia, but excessive autophagy can damage the heart during reperfusion (Matsui et al., 2007).

Mitophagy, which is the specific autophagic elimination of mitochondria, is a distinctive process that regulates the number of mitochondria and degrades damaged mitochondria (Narendra et al., 2008; Ding et al., 2011; Youle and Narendra, 2011). Similar to autophagy, mitophagy also has a bidirectional regulatory effect and may play a key role in sepsis-induced myocardial injury. Mitophagy is activated during sepsis, and Parkin-mediated mitophagy has a protective effect on sepsis-related cardiac energy metabolism disorder (Piquereau et al., 2013a). Additionally, cardiomyocyte ATP levels are increased and cardiomyocyte inflammation is reduced by rapamycin via the promotion of mitophagy, indicating the protective function of mitophagy in the myocardium during sepsis (Hsieh et al., 2011). However, the opposite notion has been suggested: mitochondrial damage in the heart is more severe in LPS-treated catalase transgenic mice, and the autophagy inhibitor 3-methyladenine can alleviate LPS-induced myocardial contractile dysfunction (Turdi et al., 2012). Therefore, the specific mechanism of autophagy requires further study.

The human aldehyde dehydrogenase (ALDH) family consists of 19 members (Vasiliou and Nebert, 2005). Within the ALDH family, ALDH2 is a mitochondrial enzyme that has the highest affinity for acetaldehyde (Vasiliou et al., 2000). ALDH2 plays a major role in protecting cells from the effects of acetaldehyde and fatty acid-derived aldehydes, such as 4-hydroxy-trans-2-nonenal (4HNE), by oxidizing them to the corresponding acid (Schnepper et al., 1991; Marchitti et al., 2008; Gomes et al., 2014). ALDH2 deficiency aggravates ethanol-induced cardiomyocyte function (Ma et al., 2010) and ALDH2 activity can reduce cardiac dysfunction (Wang et al., 2011). Similarly, ALDH2 has a bidirectional regulatory effect on autophagy. ALDH2 has a beneficial effect in myocardial ischemia/reperfusion (I/R) injury, possibly through the induction of autophagy during ischemia and a reduction of autophagy during reperfusion (Ma et al., 2011). We reported previously that ALDH2 activation can inhibit myocardial I/R injury and reduce myocardial cell apoptosis by inhibiting I/R-induced oxidative stress injury and suppressing phosphatase and tensin homolog-induced putative kinase 1 (PINK1)/Parkin-dependent mitophagy (Ji et al., 2016).

Therefore, we hypothesized that increased ALDH2 activity may have a protective effect on sepsis-induced cardiomyopathy. This effect may be realized by regulating mitophagy and, therefore, apoptosis of cardiomyocytes.

## Materials and Methods

### Animals and Treatment

All animal procedures were approved by and performed in accordance with the guidelines of the Animal User and Ethical Committees of Beijing Chao-Yang Hospital. Male C57BL/6 mice (5 weeks old) were supplied by the Shanghai SLACCAS Experimental Animal Center. Mice were housed in a temperature-controlled room under a 12 h light/dark schedule with free access to food and water. The mice were subjected to adaptive feeding for 1 week, and the experiments were performed at 6–8 weeks of age. For these experiments, 6–8 week-old mice were evaluated by echocardiography and then divided randomly into 4 groups (*n* = 6 per group): control, LPS, LPS plus Alda-1 (LPS + Alda-1; Alda-1 is an ALDH2 activator), and LPS plus ALDH2 overexpression lentivirus (LPS + ALDH2). To mimic acute endotoxemia, mice were injected intraperitoneally with 0.5 mg/kg *Escherichia coli* O55:B5 LPS dissolved in saline. Lentivirus was delivered via tail vein injection, and LPS was administered 4 weeks later. Control group mice were administered the equivalent volume of saline. Twelve hours later, all mice were euthanized to evaluate cardiac function by echocardiography. Three mice from each group were used for biological experiments and the remaining three were used for pathological experiments.

We also designed experiments to assess the effect of an ALDH2 inhibitor (CVT) or activator (Alda-1). To assess the effect of an ALDH2 inhibitor, 6–8 week-old mice were evaluated by echocardiography and then divided randomly into 4 groups (*n* = 6 per group): control, CVT, LPS, and LPS plus CVT (LPS + CVT). Similarly, to assess the effect of an ALDH2 activator, 6–8 week-old mice were evaluated by echocardiography and then divided randomly into 4 groups (*n* = 6 per group): control, Alda-1, LPS and LPS + Alda-1. For the experiments with the addition of an ALDH2 activator or inhibitor, three mice in each group were used for western blot analysis and the remaining three were used for TUNEL staining.

### Echocardiography Evaluation

After 12-h LPS treatment, the mice were anesthetized and two-dimensional M-mode transthoracic echocardiography was performed using an ultrasound system (VEVO 3100; VisualSonics, Toronto, Canada). The ejection fraction (EF), fractional shortening (FS), corrected left ventricular mass, end-diastolic left ventricular inner dimension (LVIDd), end-systolic left ventricular inner dimension (LVIDs), left ventricular posterior wall thickness at the diastolic phase (LVPWd), and left ventricular posterior wall thickness at the systolic phase (LVPWs) were analyzed. Data were recorded and analyzed blind to treatment. Results are presented as the mean ± standard error of the mean.

### Determination of Biochemical Markers of Myocardial Injury

After echocardiography, blood samples were taken. After centrifugation, the serum was used to detect biochemical changes indicating myocardial damage. Lactate dehydrogenase, creatine kinase myocardial-bound, creatine kinase, *α*-hydroxybutyrate dehydrogenase, and aspartate aminotransferase activity and blood cardiac troponin I levels were evaluated.

### Hematoxylin and Eosin Staining

Mice were euthanized and their hearts were fixed in 4% formaldehyde. Heart tissue was embedded in paraffin and sectioned. The sections were stained with hematoxylin and eosin and examined at ×400 magnification under a BX43 microscope (Olympus, Tokyo, Japan).

### Immunohistochemical Analysis

For immunohistochemical analyses, the sections were soaked in xylene to remove paraffin and dehydrated in a graded series of ethanol. For antigen retrieval, the sections were microwaved in citrate buffer for 10 min and cooled to room temperature. The sections were incubated with 3% H_2_O_2_ to block endogenous peroxidase activity. Following this, the sections were blocked with 1% bovine serum albumin at room temperature and probed with primary antibodies against tumor necrosis factor-*α* (1:100, ab9739; Abcam, Cambridge, MA) overnight at 4°C. The sections were incubated with a secondary antibody for 20 min at room temperature. Immunostaining was visualized using 3,3′-diaminobenzidine, and the sections were counterstained with hematoxylin and dehydrated in a graded series of ethanol. Finally, the sections were mounted with neutral gum and observed and photographed at ×400 magnification under an optical BX43 microscope (Olympus).

### Measurement of Aldehyde Dehydrogenase 2 Activity

ALDH2 activity was measured in 33 mmol/L sodium pyrophosphate containing 0.8 mmol/L NAD+, 15 mmol/L propionaldehyde, and 0.1 ml protein extract. Propionaldehyde, the substrate of ALDH2, was oxidized in propionic acid, and NAD+ was reduced to NADH to estimate ALDH2 activity. NADH was determined by spectrophotometric absorbance at 340 nm.

### Western Blotting

Target proteins in cardiac tissue homogenates were extracted using a whole protein extraction kit (KeyGen Biotech, Nanjing, China). Protein concentration was determined using a BCA protein content detection kit (KeyGen Biotech). The target proteins were separated by 10%, 12%, or 15% sodium dodecyl sulfate-polyacrylamide gel electrophoresis. They were then electrotransferred to nitrocellulose membranes. The membranes were blocked with 5% nonfat milk and incubated at 4°C overnight with the following primary antibodies: anti-4HNE (1:3,000, ab46545; Abcam), anti-ALDH2 (1:1,000, 15,310-1-ap; Proteintech, Rosemont, IL), anti-P62 (1:1,000, 18,420-1-ap; Proteintech), anti-PINK1 (1:500, 23,274-1-ap; Proteintech), anti-Parkin (1:1,000, 14,060-1-ap, Proteintech), anti-BCL2-interacting protein 3 (BNIP3, 1:1,000, ab109362; Abcam), anti-FUN14 domain-containing 1 (FUNDC1, 1:1,000, ab224722; Abcam), anti-microtubule-associated protein 1 light chain 3 beta (LC3B, 1:500, 18,725-1-ap; Proteintech), anti-caspase 3 (1:500, 19,677-1-ap; Proteintech), anti-BAX (1:3,000, 50599-2-Ig; Proteintech), anti-BCL2 (1:1,000, 12,789-1-ap; Proteintech), and anti-glyceraldehyde 3-phosphate dehydrogenase (1:10,000, KGAA002; KeyGen Biotech), which was used as a loading control. Peroxidase-conjugated secondary antibodies were incubated with the membranes for 2 h at room temperature. Signals were visualized using an enhanced chemiluminescence detection system (G:BOX ChemiXR5; Syngene International, Bangalore, India). Protein bands were assessed and quantified using Gel-Pro32.

### Transmission Electron Microscopy

Mitochondria of cardiomyocytes were observed using transmission electron microscopy (TEM). Cardiac tissue was fixed with glutaraldehyde and treated with osmium tetroxide. Tissue blocks were dehydrated through a graded series of ethanol and embedded. Thin sections (50–60 nm) were cut on an ultramicrotome and stained with lead citrate/uranyl acetate. Images were acquired using a JEM-1400 (JEOL Ltd., Tokyo, Japan).

### Measurement of Plasma Malondialdehyde

An MDA assay kit (Nanjing Jiangcheng Bioengineering Institute, Nanjing, China) was used to measure malondialdehyde (MDA) production. Optical density was measured with a Spectramac M3 multiscan spectrum instrument (Molecular Devices, LLC, Sunnyvale, CA) at 532 nm.

### Measurement of Plasma Superoxide Dismutase

SOD activity was assessed using an assay kit (Nanjing Jiancheng Bioengineering Institute) at 37°C for 20 min. Optical density was measured using an EL-x800 microplate reader (BioTek Instruments, Winooski, VT).

### Measurement of Mitochondrial Membrane Potential

Myocardial tissues were sheared to extract high-purity mitochondria using a mitochondrial extraction kit (KeyGen Biotech), and MMP was analyzed with an assay kit (KeyGen Biotech). Prepared working staining fluid (180 μL) was added to 20 μL purified mitochondria to give a total protein mass of 10–100 ug and incubated in a 5% CO_2_ incubator at 37°C for 10–20 min. A Spectramac M3 multiscan spectrum instrument (Molecular Devices) was used to detect JC-1 polymer (excitation and emission wavelengths were 525 and 590 nm, respectively) and JC-1 monomer (excitation and emission wavelengths were 490 and 530 nm, respectively).

### Measurement of Apoptosis

An Apoptosis Assay Kit (KeyGen Biotech) was used for TUNEL staining. The cells were observed and photographed under a BX43 fluorescence microscope (Olympus).

### Data Analysis

Data are presented as the mean ± standard error of the mean. Multiple groups were compared using one-way analysis of variance followed by Tukey’s *post hoc* test. All analyses were performed using Prism 5 (GraphPad Software, Inc., San Diego, CA). *P* < 0.05 or *P* < 0.01 was considered statistically significant.

## Results

### Aldehyde Dehydrogenase 2 Alleviates Lipopolysaccharide-Induced Myocardial Injury

**FIGURE 1:**
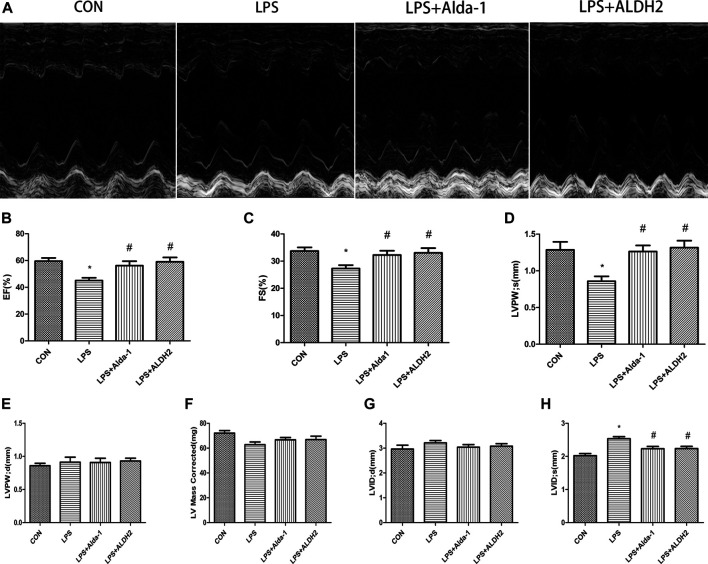
Cardiac function in lipopolysaccharide (LPS)-treated mice. **(A)** Representative echocardiographic images from four mouse groups. **(B)** Ejection fraction (EF). **(C)** Fractional shortening (FS). **(D)** Left ventricular posterior wall thickness in systole (LVPWs). **(E)** Left ventricular posterior wall thickness in diastole (LVPWd) **(F)** Left ventricular mass. **(G)** End-diastolic left ventricular internal diameter (LVIDd). **(H)** End-systolic left ventricular internal diameter (LVIDs). Data are the mean ± standard error of the mean. *n* = 6 mice/group. Statistical analyses were performed by one-way analysis of variance, followed by Tukey’s *post hoc* test for multiple comparisons. *p < 0.05 vs. control (CON); #*p* < 0.05 vs. LPS.

**FIGURE 2:**
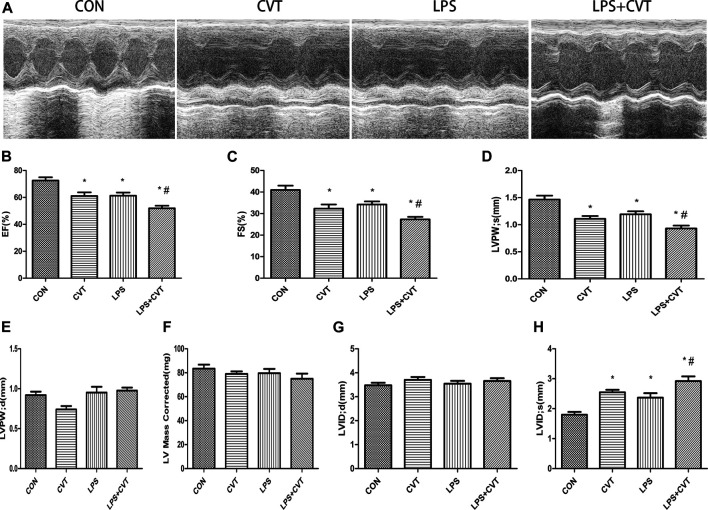
Cardiac function in lipopolysaccharide (LPS)-treated mice. **(A)** Representative echocardiographic images from four mouse groups. **(B)** Ejection fraction (EF). **(C)** Fractional shortening (FS). **(D)** Left ventricular posterior wall thickness in systole (LVPWs). **(E)** Left ventricular posterior wall thickness in diastole (LVPWd). **(F)** Left ventricular mass. **(G)** End-diastolic left ventricular internal diameter (LVIDd). **(H)** End-systolic left ventricular internal diameter (LVIDs). Data are the mean ± standard error of the mean. *n* = 6 mice/group. Statistical analyses were performed by one-way analysis of variance, followed by Tukey’s *post hoc* test for multiple comparisons. *p < 0.05 vs. control (CON); #*p* < 0.05 vs. LPS.

Echocardiography images are shown in Figure 1A. Compared with LPS alone, LPS + Alda-1 and LPS + ALDH2 increased the EF, FS, and LVPWs (Figures 1B–D). There were no significant differences in LVPWd, left ventricular mass, and LVIDd among the groups (Figures 1E–G). In contrast, LPS + Alda-1 and LPS + ALDH2 reduced LVIDs (Figure 1H). On the other hand, other echocardiography images are shown in Figure 2A. Compared with LPS alone, LPS + CVT decreased the EF, FS, and LVPWs (Figures 2B–D). There were no significant differences in LVPWd, left ventricular mass, and LVIDd among the groups (Figures 2E–G). In contrast, LPS + CVT increased LVIDs (Figure 2H). Therefore, ALDH2 activation or overexpression improved myocardial function in LPS-treated mice. On the contrary, ALDH2 inhibition impaired myocardial function in LPS-treated mice.

**FIGURE 3:**
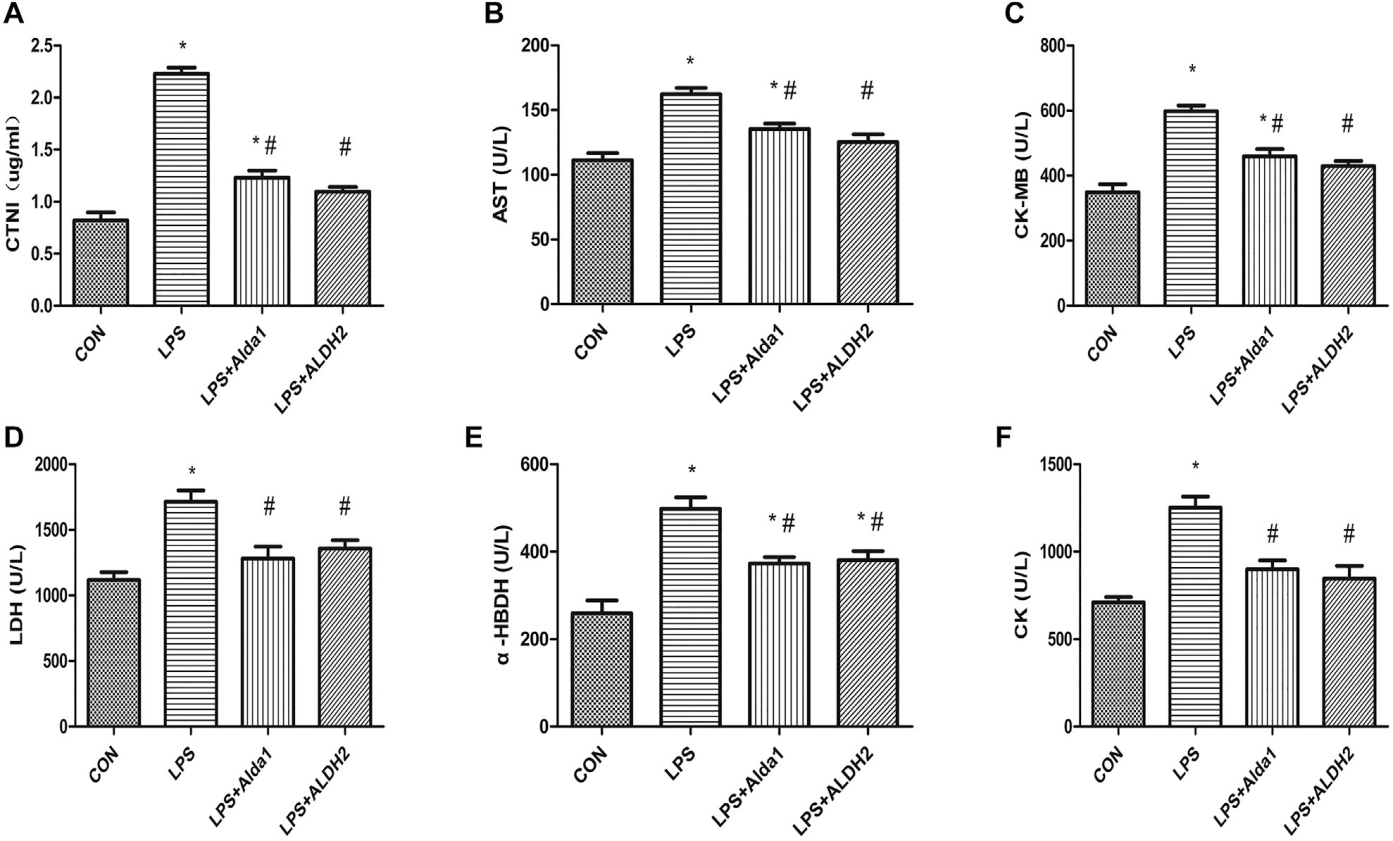
Biochemical markers of myocardial injury in lipopolysaccharide (LPS)-treated mice. **(A)** Cardiac troponin I (CTNI) levels. **(B)** Aspartate aminotransferase (AST), **(C)** Creatine kinase myocardial-bound (CK-MB), **(D)** Lactate dehydrogenase (LDH), **(E)** α-Hydroxybutyrate dehydrogenase (*α*-HBDH), and **(F)** CK activity in serum. Data are the mean ± standard error of the mean. *n* = 3 mice/group. Statistical analyses were performed by one-way analysis of variance, followed by Tukey’s *post hoc* test for multiple comparisons. *p < 0.05 vs. control (CON); #*p* < 0.05 vs. LPS.

To examine the role of ALDH2 in LPS-induced myocardial damage, we evaluated the concentration of cardiac troponin I and the activity of enzymes that are markers of myocardial injury. The concentration of cardiac troponin I was increased in the LPS-induced group compared with the control group, and this increase was inhibited by ALDH2 activation or overexpression (Figure 3A). Similarly, the activity of aspartate aminotransferase, creatine kinase myocardial-bound, lactate dehydrogenase, *α*-hydroxybutyrate dehydrogenase, and creatine kinase was increased in the LPS-induced group compared with the control group, and these increases in activity were also inhibited by ALDH2 activation or overexpression (Figures 3B–F). Therefore, ALDH2 activation or overexpression conferred cardioprotection.

**FIGURE 4:**
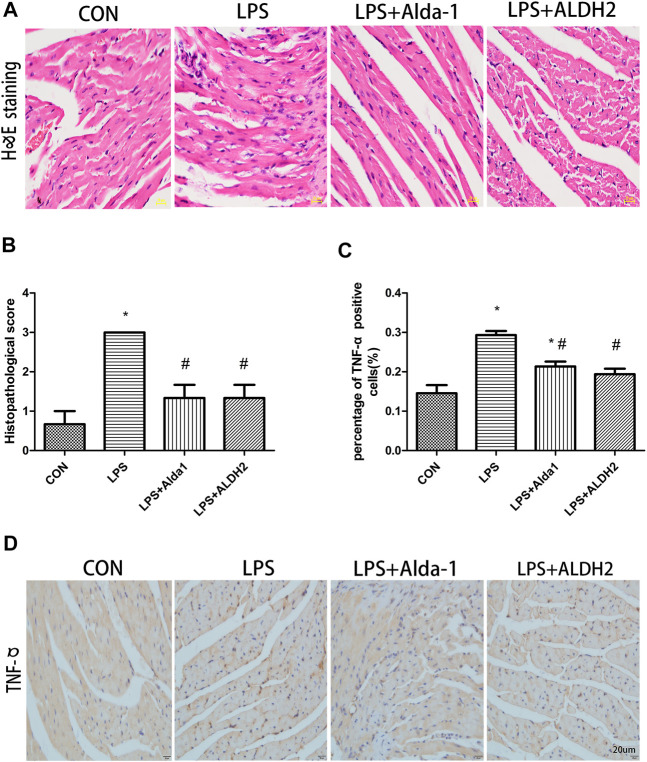
Heart tissue pathology and inflammation in lipopolysaccharide (LPS)-treated mice. **(A)** Hematoxylin and eosin staining of heart tissue. Magnification, ×400. Scale bar: 20 µm. **(B)** Histopathological scores of heart tissue. **(C)** Quantification of tumor necrosis factor (TNF)-α-positive cells detected by immunostaining. **(D)** TNFα expression was examined by immunohistochemical staining. Scale bar: 20 µm. Data are the mean ± standard error of the mean. *n* = 3 mice/group. Statistical analyses were performed by one-way analysis of variance, followed by Tukey’s *post hoc* test for multiple comparisons. *p < 0.05 vs. control (CON); #*p* < 0.05 vs. LPS.

When myocardial inflammatory cell infiltration was assessed in LPS-treated mice, compared with the control group, LPS stimulated greater cellular degeneration, tissue edema, hyperemia, and inflammatory cell infiltration. After treatment with the ALDH2 activator Alda-1 or the ALDH2 overexpression virus, cellular degeneration, tissue edema, hyperemia, and inflammatory cell infiltration were less evident than in the LPS-treated group (Figures 4A,B). Similarly, the level of tumor necrosis factor-α in heart tissue was significantly increased after LPS treatment, and this increase was markedly inhibited by ALDH2 activation or overexpression (Figures 4C,D). Therefore, ALDH2 activation or overexpression reduced myocardial inflammatory responses.

### 4-Hydroxy-Trans-2-Nonenal Accumulation, Aldehyde Dehydrogenase 2 Expression and Activity, Ultrastructural Changes, and Mitophagy in Lipopolysaccharide-Treated Mice

**FIGURE 5:**
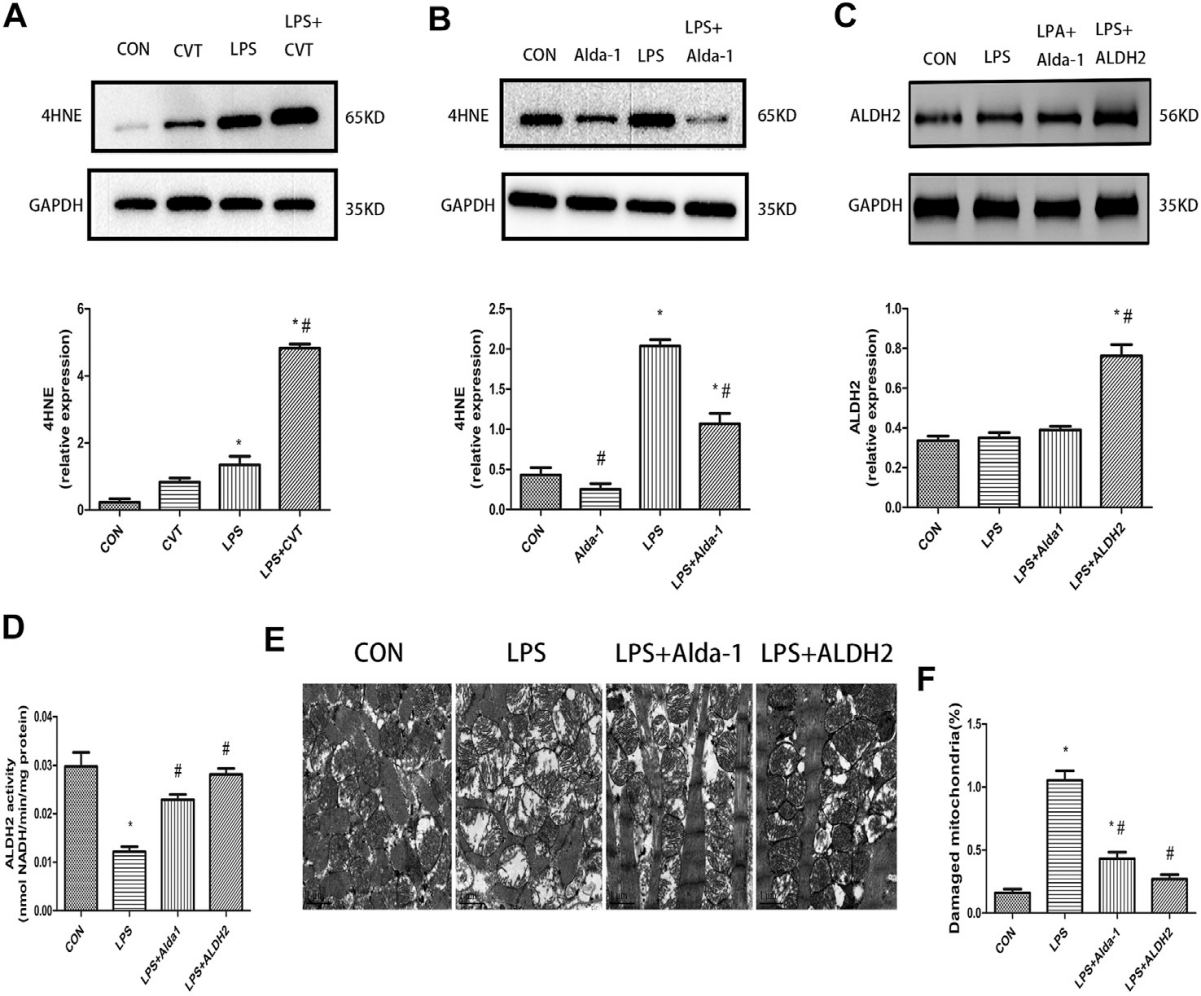
4-Hydroxy-trans-2-nonenal (4HNE) accumulation, aldehyde dehydrogenase 2 (ALDH2) activity and expression, and ultrastructural changes in lipopolysaccharide (LPS)-treated mice **(A,B)** Representative western blots and quantification analysis of 4HNE and glyceraldehyde 3-phosphate dehydrogenase (GAPDH; loading control). **(C)** Representative western blots and quantification analysis of ALDH2 and GAPDH (loading control). **(D)** Quantification of ALDH2 enzymatic activity. **(E)** Representative transmission electron microscopy (TEM) images from four mouse groups. Scale bar: 1 µm. **(F)** Quantification analysis of damaged mitochondria. Data are the mean ± standard error of the mean. *n* = 3 mice/group. Statistical analyses were performed by one-way analysis of variance, followed by Tukey’s *post hoc* test for multiple comparisons. *p < 0.05 vs. control (CON); #*p* < 0.05 vs. LPS.

We evaluated the lipid peroxidation end product 4HNE, which is a key ALDH2 substrate, in the myocardium. We found that LPS treatment alone increased 4HNE accumulation compared with the control group. This increase was enhanced by ALDH2 inhibition (Figure 5A) but was mitigated by ALDH2 activation (Figure 5B).

LPS treatment had no effect on ALDH2 expression (Figure 5C). However, LPS significantly decreased ALDH2 activity, and this decrease was rescued by ALDH2 activation or overexpression (Figure 5D).

To determine the morphological changes of mitochondria induced by LPS, we examined mitochondrial ultrastructure by TEM. After LPS treatment, the fracture and disappearance of mitochondrial cristae and many vacuoles were detected. These changes represent mitochondrial damage and were alleviated by ALDH2 activation or overexpression (Figures 5E,F).

**FIGURE 6:**
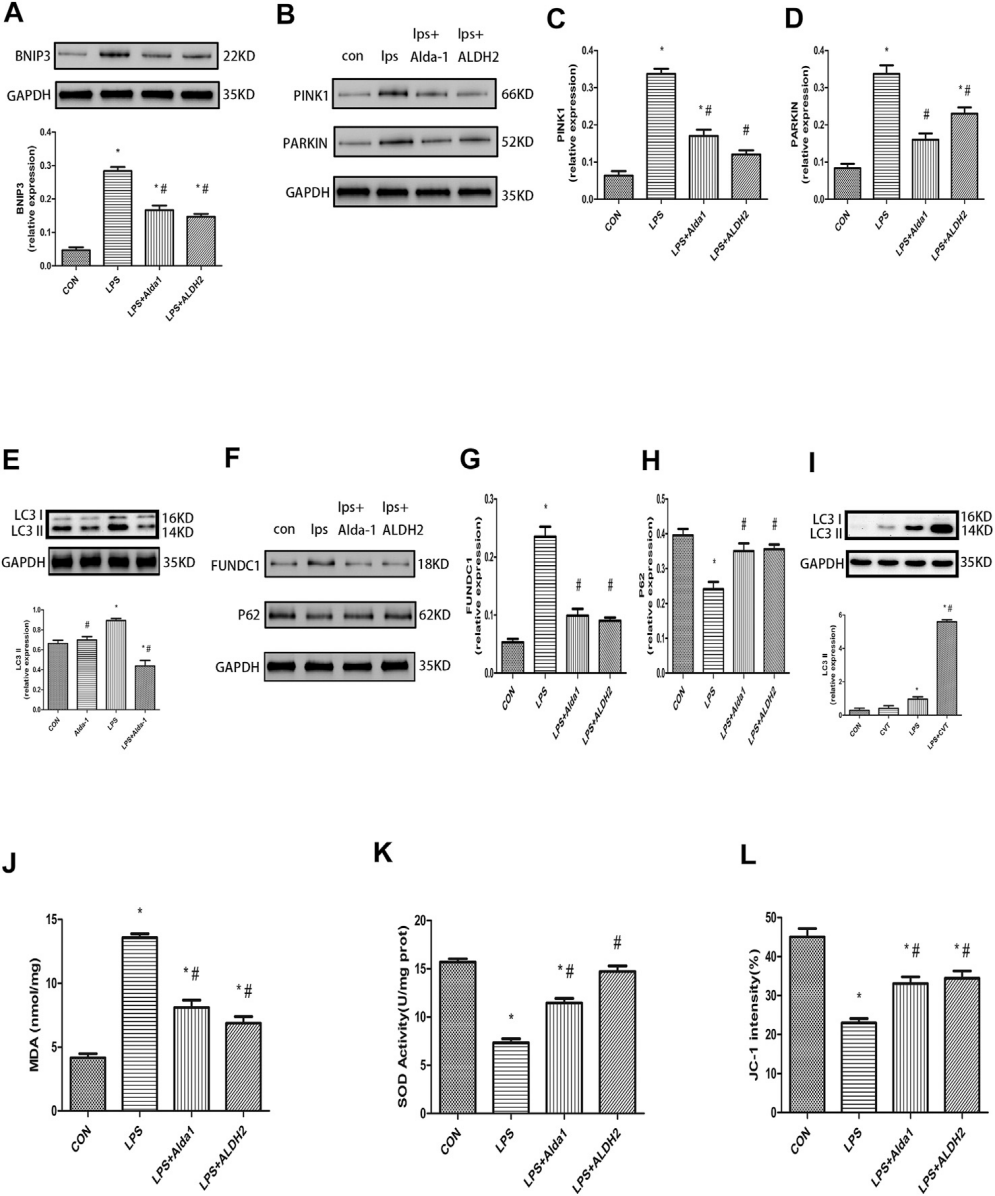
Mitophagy, malondialdehyde (MDA), superoxide dismutase (SOD) activity, and mitochondrial membrane potential (MMP) in lipopolysaccharide (LPS)-treated mice. **(A)** Representative western blots and quantification analysis of BCL2-interacting protein 3 (BNIP3) and glyceraldehyde 3-phosphate dehydrogenase (GAPDH; loading control). **(B)** Representative western blots of phosphatase and tensin homolog-induced putative kinase 1 (PINK1), Parkin, and GAPDH (loading control). Quantification of **(C)** PINK1 and **(D)** Parkin. **(E)** Representative western blots and quantification of microtubule-associated protein 1 light chain 3 beta (LC3B) and GAPDH (loading control). **(F)** Representative western blots of FUN14 domain-containing 1 (FUNDC1), P62, and GAPDH (loading control). Quantification of **(G)** FUNDC1 and **(H)** P62. **(I)** Representative western blots and quantification of LC3B and GAPDH (loading control). **(J)** MDA levels in plasma. **(K)** SOD activity in plasma. **(L)** Quantification of JC-1 intensity for MMP. Data are the mean ± standard error of the mean. *n* = 3 mice/group. Statistical analyses were performed by one-way analysis of variance, followed by Tukey’s *post hoc* test for multiple comparisons. *p < 0.05 vs. control (CON); #*p* < 0.05 vs. LPS.

The PINK1/Parkin pathway is essential for the control of mitophagy. Therefore, to explore the mechanism of mitophagy in LPS-induced cardiomyocyte injury, we examined the levels of key mitophagy proteins, including BNIP3, PINK1, Parkin, LC3B, FUNDC1, and P62. ALDH2 activation or overexpression suppressed the LPS-induced increase of BNIP3, PINK1, Parkin, LC3B, and FUNDC1 levels (Figures 6A–G). In contrast, ALDH2 activation or overexpression ameliorated the LPS-induced reduction of P62 expression (Figures 6F,H), a ubiquitin and LC3-binding protein that is a selective autophagy substrate and can be degraded by autophagosomes (Pankiv et al., 2007; Ichimura et al., 2008). Conversely, ALDH2 inhibition promoted the LPS-induced increase of LC3B (Figure 6I). These results indicated that ALDH2 can play a prominent role in mitophagy regulation during myocardial LPS injury. ALDH2 activation or overexpression protected cardiomyocytes against LPS injury by suppressing PINK1/Parkin-dependent mitophagy via a reduction in the accumulation of 4HNE.

### Malondialdehyde, Superoxide Dismutase Activity, Mitochondrial Membrane Potential, and Apoptosis in Lipopolysaccharide-Treated Mice

**FIGURE 7:**
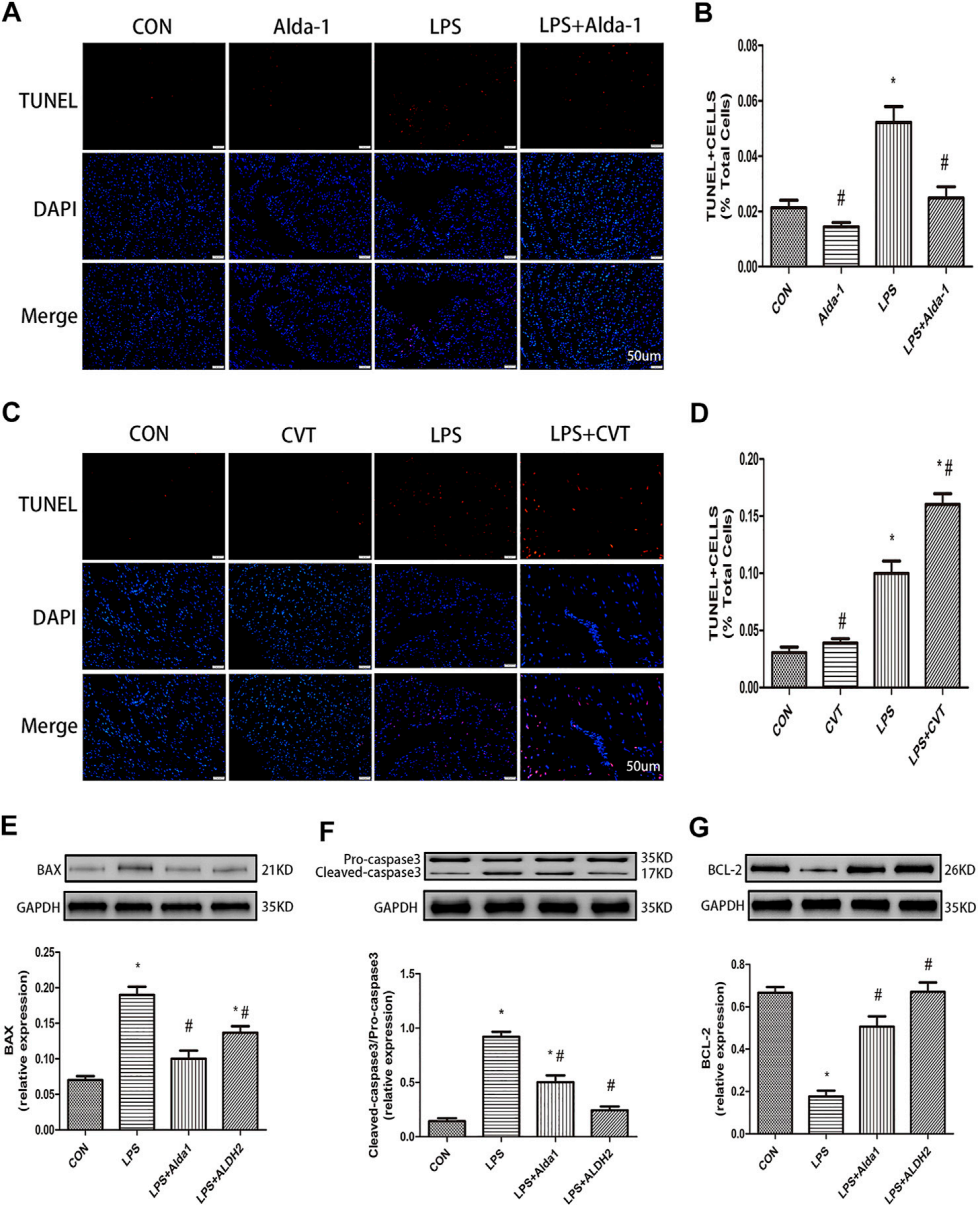
Apoptosis in lipopolysaccharide (LPS)-treated mice. **(A)** TUNEL staining for myocardial cell apoptosis. **(B)** Ratio of apoptosis. Representative TUNEL staining images are shown (magnification, ×400). Scale bar: 20 µm **(C)** TUNEL staining for myocardial cell apoptosis. **(D)** Ratio of apoptosis. Representative TUNEL staining images are shown (magnification, ×400). Scale bar: 20 µm. Representative western blots and quantification of **(E)** BAX, **(F)** caspase 3, **(G)** BCL2, and glyceraldehyde 3-phosphate dehydrogenase (GAPDH; loading control). Data are the mean ± standard error of the mean. *n* = 3 mice/group. Statistical analyses were performed by one-way analysis of variance, followed by Tukey’s post hoc test for multiple comparisons. *p < 0.05 vs. control (CON); #*p* < 0.05 vs. LPS.

To explore the role of oxidative stress in ALDH2-mediated cardioprotection, we evaluated MDA levels, SOD activity, MMP, and apoptosis in LPS-treated mice. ALDH2 activation or overexpression markedly decreased MDA levels in LPS-treated mice (Figure 6J). However, ALDH2 activation or overexpression significantly alleviated the decreased SOD activity and MMP in LPS-treated mice (Figures 6K,L). LPS treatment alone induced cardiomyocyte apoptosis, which was alleviated by ALDH2 activation or overexpression (Figures 7A,B). Conversely, this effect was enhanced by ALDH2 inhibition (Figures 7C,D). The expression levels of apoptosis-related proteins were measured, and LPS treatment alone increased the levels of BAX and caspase 3, and these increases were reduced by ALDH2 activation or overexpression (Figures 7E,F). Accordingly, BCL2 expression was increased by ALDH2 activation or overexpression (Figure 7G). These findings support a central role for ALDH2 in LPS-induced myocardial injury. ALDH2 activation or overexpression ameliorated the LPS-induced increase in apoptosis and reduction in MMP by suppressing oxidative stress.

## Discussion

Sepsis is caused by a dysregulated host response to infection (Taeb et al., 2017). LPS, which is the major component of the outer membrane of Gram-negative bacteria, is crucial for the viability and virulence of bacteria (Whitfield and Trent, 2014) and for the induction of innate immune responses and sepsis (Bryant et al., 2010). Autophagy is a dynamic catabolic cellular process whose the most pivotal characteristic is the formation of a double membrane-bound compartment known as the autophagosome (Nakatogawa et al., 2009; Klionsky et al., 2016). There are two types of autophagy: non-selective autophagy and selective autophagy; mitophagy is a form of selective autophagy (Youle and Narendra, 2011). We previously confirmed that ALDH2 can inhibit myocardial ischemia/reperfusion injury by regulating mitophagy (Ji et al., 2016). Many studies have shown that mitophagy plays a prominent role in sepsis-associated myocardial injury (Hsieh et al., 2011; Turdi et al., 2012; Piquereau et al., 2013b). One study demonstrated excessive endoplasmic reticulum stress and mitophagy levels in an animal model of sepsis, which were significantly alleviated in ALDH2 transgenic mice (Pang et al., 2019a). In the present study, we found that ALDH2 activation or overexpression could counteract sepsis-associated cardiac injury *in vivo* by downregulating mitophagy via the mitigation of 4HNE accumulation and MDA formation. On the contrary, ALDH2 inhibition can damage the myocardium *in vivo* by upregulating autophagy via the accumulation of 4HNE. In conclusion, our results indicated that ALDH2 had a pivotal positive effect in mediating cardiac protection following LPS administration.

Mitochondria are crucial for energy production and reactive oxygen species (ROS) metabolism (Starkov, 2008; Kowaltowski et al., 2009). Interestingly, mitochondria can serve as a “powerplant” to supply energy for the cardiomyocytes. Oxidative stress, the release of inflammatory factors, and dysregulated autophagy in sepsis cause mitochondrial dysfunction, thereby accelerating the development of myocardial dysfunction (Tan et al., 2019). Oxidative stress is one of the key points in our study. An imbalance between the generation of harmful ROS and antioxidant defenses is a crucial factor in the oxidative stress response (Yan et al., 2013). A major cytotoxic product of lipid peroxidation is 4HNE, which is indirectly related to ROS (Esterbauer et al., 1991; Schneider et al., 2001). Moreover, 4HNE has a role in oxidative stress-related diseases, including cardiovascular diseases, neurodegenerative diseases, liver diseases, metabolic syndrome, and cancer (Dalleau et al., 2013). However, myocardial injury induced by ischemia/reperfusion and doxorubicin is alleviated by ALDH2 overexpression or activation via the regulation of 4HNE (Ma et al., 2011; Sun et al., 2014). Similarly, our present study demonstrated that ALDH2 activation or overexpression could mitigate cardiac injury by alleviating 4HNE accumulation in LPS-induced cardiac injury. Conversely, ALDH2 inhibition aggravated cardiac injury by increasing the accumulation of 4HNE in LPS-induced cardiac injury. It has been proposed that the process of apoptosis will finally cause a decrease in the number of cardiomyocytes, and if the number of cardiomyocytes is reduced to a certain extent, it will result in myocardial dysfunction (Tan et al., 2019). Moreover, we observed LPS-induced reductions in ALDH2 activity and MMP depolarization that were associated with increased apoptosis of myocardial cells and suppressed cardiac function. However, protein kinase C does not exert a neuroprotective effect in ALDH2-knockdown rats after stroke (Guo et al., 2013). Another study also showed that ALDH2 repression is associated with a poor prognosis in patients with lung adenocarcinoma (Li et al., 2019). Therefore, the restoration of ALDH2 activity or increased ALDH2 expression and the elimination of oxidative stress may be an effective treatment for LPS-induced myocardial dysfunction.

To protect cells, damaged mitochondria are sequestered by autophagosomes and degraded before apoptosis can be triggered (Kubli and Gustafsson, 2012). The PINK1/Parkin pathway is critical for the regulation of mitophagy (Zhao et al., 2019), and PINK1 is a crucial initiator of mitophagy (Wang et al., 2019). Reduced MMP leads to the accumulation of PINK1, and the E3 ubiquitin ligase Parkin is subsequently recruited by PINK1 to mitochondria (Kubli and Gustafsson, 2012). However, it is unclear exactly how PINK1 recruits Parkin. Furthermore, ROS-induced mitochondrial damage may be a prominent upstream activator of mitophagy (Wang et al., 2012). BNIP3 is an inducer of mitophagy, and BNIP3-mediated autophagy involves the translocation of Parkin to mitochondria (Lee et al., 2011). FUNDC1, a mitochondrial outer-membrane protein, is a mitophagy receptor that regulates mitochondrial dynamics and mitophagy (Liu et al., 2012; Chen et al., 2016). Furthermore, FUNDC1 has been implicated in Parkin-independent mitophagy and interacts with LC3 to induce mitophagy (Liu et al., 2012). P62 protein, also called sequestosome 1, binds to LC3 during mitophagy (Pankiv et al., 2007; Kubli and Gustafsson, 2012) and to ubiquitinated mitochondrial proteins (Kubli and Gustafsson, 2012). Many studies have shown that the levels of BNIP3, PINK1, Parkin, LC3B, and FUNDC1 are associated with mitophagic activity (Chen et al., 2014; Hu et al., 2016; Wei et al., 2018; Zhao et al., 2020). In the present study, we showed the increased levels of MDA and 4HNE and activation of the PINK1/Parkin pathway in LPS-induced myocardial dysfunction. A previous study revealed likely roles for ALDH2 in the inflammatory response, immunity, and organ dysfunction in sepsis (Pang et al., 2019b). We also provided evidence that ALDH2 activation with the activator Alda-1 or ALDH2 overexpression downregulated the PINK1/Parkin pathway and suppressed subsequent biochemical injury, inflammation, apoptosis, and myocardial dysfunction. On the other hand, we showed that ALDH2 inhibition upregulated the autophagy pathway and promoted subsequent cell apoptosis and myocardial dysfunction.

Mitochondrial function and quality control are especially important for a healthy heart (Piquereau et al., 2013b; Pan et al., 2018). Mitochondrial dysfunction occurs during the pathophysiology of septic cardiomyopathy. Many factors such as inflammation, oxidative stress, and dysregulated autophagy during sepsis can have a negative impact on mitochondrial function and accelerate the progress of myocardial dysfunction (Tan et al., 2019). Many studies have shown that mitophagy is protective for the cardiovascular system, and treatment for mitophagy is beneficial for cardiovascular disease (Shires and Gustafsson, 2015; Zhang et al., 2018). In LPS-induced myocardial injury, the upregulation of tumor susceptibility gene 101 protects against LPS-triggered myocardial injury by promoting Parkin-mediated mitophagy (Essandoh et al., 2019). Additionally, mitochondrial uncoupling protein 2 may play a salutary role in LPS-induced cardiomyocyte apoptosis by increasing the levels of the mitophagy proteins Beclin 1 and LC3β (Pan et al., 2018). In response to LPS-mediated mitochondrial damage, deletion of mammalian Ste20-like kinase 1 (Mst1) activates mitophagy to protect mitochondria. However, repressing Parkin-mediated mitophagy abolishes the positive influence of Mst1 deletion on mitochondrial protection and myocardial cell viability (Shang et al., 2020). Dexmedetomidine (DEX) alleviates LPS-induced apoptosis and the inflammatory response of macrophages via PINK1-mediated mitophagy (Wang et al., 2019). DEX increases the levels of autophagy and mitophagy proteins, such as Beclin 1, LC3II, and PINK1. Furthermore, DEX reduces the levels of ROS and apoptosis. Through the above mechanism, DEX was shown to improve acute LPS-induced kidney injury (Zhao et al., 2020). Similarly, PINK1/Parkin-mediated mitophagy might have a positive effect on acute LPS-induced kidney injury (Dai et al., 2019). Further research has found that in PINK1 or PARK2 knockout mice, sepsis increases more serious renal cell apoptosis and causes kidney injury (Wang et al., 2021). In contrast, targeting BCL2 overexpression mitigates LPS-induced acute lung injury by inhibiting mitophagy and apoptosis, and ultimately improves the survival of mice (Zhang et al., 2020). Another study also notes that in LPS-induced myocardial model, ALDH2 transgene can alleviate mitophagy and apoptosis (Pang et al., 2019b). Our analysis of mitochondrial ultrastructure indicated that LPS could damage cardiac mitochondria. One study proved that mitophagy can impair mitochondrial function (Tyrrell et al., 2020). We also provided evidence that the activation of mitophagy was harmful in LPS-induced myocardial injury. Most importantly, ALDH2 activation or overexpression alleviated LPS-induced injury in mice by inhibiting mitophagy. ALDH2 inhibition exacerbated LPS-induced myocardial injury by promoting autophagy. With excess oxidative stress, the PINK1/Parkin pathway may be activated excessively as a reaction to LPS injury to cause myocardial cell death. ALDH2 activation or overexpression alleviated LPS-induced apoptosis of myocardial cells, in part, by suppressing the over-activation of mitophagy. ALDH2 inhibition can exacerbate LPS-induced apoptosis of myocardial cells, in part, by promoting the over-activation of autophagy.

Additional studies are needed to understand completely how mitophagy activity and mitochondrial morphology/function are regulated under LPS stimulation and whether the myocardium can be improved by therapeutically altering ALDH2 activity and expression.

## Conclusion

ALDH2 inhibited excessive mitophagy and increased the survival of LPS-induced cardiomyocytes by reducing oxidative stress levels. These findings improve our understanding of the ALDH2 mechanisms responsible for protection against LPS-induced myocardial injury. Given that ALDH2 is cardioprotective in a mouse LPS model, ALDH2 is a potential therapeutic target for septic myocardial injury.

## Data Availability

The original contributions presented in the study are included in the article/Supplementary Material, further inquiries can be directed to the corresponding authors.
